# Characterization, antioxidant and anticoagulant properties of exopolysaccharide from marine microalgae

**DOI:** 10.1186/s13568-022-01365-2

**Published:** 2022-03-03

**Authors:** Zahra Mousavian, Maliheh Safavi, Farzaneh Azizmohseni, Mahnaz Hadizadeh, Saeed Mirdamadi

**Affiliations:** Department of Biotechnology, Iranian Research Organization for Science and Technology (IROST), P. O. Box 3353-5111, Tehran, Iran

**Keywords:** Marine microalgae, Sulfated exopolysaccharide, Anticoagulant, Antioxidant

## Abstract

The sulfated exopolysaccharide extracted from marine microalgae attracted considerable attention from both the nutraceutical and pharmaceutical industries. In the present study biomass of five marine microalgae were screened to find strains with high capacity for the production of sulfated exopolysaccharides. The anticoagulant and antioxidant activities of extracted sulfated polysaccharides were evaluated using activated partial thromboplastin time (aPTT), prothrombin time (PT), DPPH and ABTS assays, respectively. The sulfated polysaccharides extracted from *Picochlorum* sp. showed a strong DPPH scavenging effect with 85% antioxidant activity**.** The sulfated polysaccharides of *Chlorella sorokiniana*, *Chlorella* sp. (L_2_) and *Chlorella *sp. (D_1_) scavenged more than 90% of the ABTS radicals. However, the sulfated polysaccharide extracted from *Chlorella sorokiniana*, and *Chlorella* sp. (*N4*) showed anticoagulant properties. The dual anticoagulant-antioxidant activities in *Chlorella sorokiniana* could be explained by the combination of various factors including sulfate content and their binding site, monosaccharide residue and glycoside bond which are involved in the polysaccharide’s bioactivity.

## Keypoints

- Sulfated exopolysaccharides (sPS) were extracted from marine green microalgae by the heated acid extraction method.
- sPS with the higher sulfate/sugar ratio presented potent ABTS radical scavenging activity.
- Some of the sPS revealed anticoagulant effects in activated partial thromboplastin time (aPTT) and prothrombin time (PT) assays.

## Introduction

There is a remarkable focus on the development of value-added functional food products with health-promoting properties and improved nutrition from the new natural marine resources (Galasso et al. 2019; Bernaerts et al. 2019). The wide diversity in the biochemical composition of marine organisms represents a rich source of valuable metabolites. Marine valuable bioactive compounds such as microalgae exopolysaccharides (EPS) are known as novel and interesting resources for the pharmaceutical and food industry (Liu et al. 2018; Zhang et al. 2019). The various biological activity of EPS is due to a complex interaction of multiple structural patterns including molecular weight, the sugar residue composition, types of glycosidic bonds, the monosaccharides nature, and the presence of some non-sugar units (sulfates, methyl, organic acid, amino acids, or amine) in the polysaccharide backbone (Berthon et al. 2017; Pierre et al. 2019). Certainly, the most common structural pattern of EPS consists of sulfate esters attached to glycosides and their sulfation level play an important role in biological applications (Zhang et al. 2019). Sulfate content between the sulfated polysaccharides (sPS) extracted from the various strains of microalgae are different (De Jesus Raposo et al. 2013). The content of sulfate sPS has a strong effect on physio-chemical characterization and biological activity (Zhang et al. 2019; Jiao et al. 2011).

Recently, the importance of microalgal sPS in cosmetics, food, medical and pharmacological industries has grown significantly (Zhang et al. 2019; Minhas et al. 2016). They have shown antibacterial, antiviral (De Jesus Raposo et al. 2013; Wu et al. 2013), anti-inflammatory (Wu et al. 2015), antioxidant, anti-tumor and lowering cholesterol activities (Bernaerts et al. 2019; Barkia et al. 2019; Amaro et al. 2013). They are used to block the adhesion of pathogenic microorganisms such as *Helicobacter pylori* on epithelial cells as an anti-adhesive agent (Guzman-Murillo and Ascencio 2000; Ascencio et al. 1993).

The pharmaceutical and food industries have shown rapid interest in the development of natural, safe and new multi-target bioactive compounds like antioxidants and anticoagulants especially of marine origin (Chu 2011; Burg and Oshrat 2015). Marine algal sPS possess potent antioxidant activities through scavenging effects on the free radicals with direct or indirect interaction. In the continuing search for new and natural resources of bioactive compounds for anticoagulant activity, microalgae sulfated polysaccharides with hemi-ester sulfate group in sugar units have been introduced. Algal sPS have an anticoagulant activity similar to heparin as a natural highly sulfated mucopolysaccharide (Mišurcová et al. 2015).

The anionic nature of sPS due to the existence of the sulfate group makes them suitable candidates for bio-applications such as antioxidant and anticoagulant agents. Microalgae sPS as a glycosaminoglycan containing sulfate ester in their various sugar units can be an interesting candidate for medical applications (Marques et al. 2016).

In the present study, the sPS production and characterization in terms of yield, total carbohydrate and sulfate contents of the five isolated microalgae strains from the Persian Gulf are described. Upon our knowledge, this study represents the first description of isolated microalgal strains [*Chlorella sorokiniana*, *Chlorella *sp. (L_2_), *Chlorella *sp. (D_1_)_,_ *Chlorella *sp. (N_4_) and *Picochlorum *sp.] from these habitats for sPS production. The strains containing the sPS with the most potent antioxidant and anticoagulant activities have been evaluated and presented.

## Material and method

### Material and chemical

Sodium nitrate, calcium chloride dehydrates, magnesium sulfate heptahydrate, potassium phosphate, monopotassium phosphate, sodium chloride, boric acid, EDTA, zinc sulfate heptahydrate, chloride tetrahydrate, molybdenum trioxide, copper (II) sulfate pentahydrate and cobalt (II) nitrate hexahydrate were obtained from Sigma-Aldrich, Dorset, UK. 2,2′-azino-bis(3-ethylbenzthiazoline-6-sulfonate) (ABTS), 1,1-diphenyl-2 picrylhydrazyl (DPPH), were purchased from Sigma Chemical Co. (St. Louis, MO, USA). Commercial kits for aPTT and PT tests were purchased from Fisher Scientific, Canada. Heparin sodium was obtained from Hi-Media Laboratories, Mumbai, India.

### Cultivation of microalgal strain

Five microalgae strains (L_1_, N_4_, D_1_, L_2_, S_1_) used in this study were isolated from the Persian Gulf and the Qeshm Island (26°32 N, 53°56 E), at the southern of Iran and deposited in Persian Type Culture Collection (PTCC) as NO. M8011, M8013, M8010, M8012, M6032, respectively. The microalgae strains were cultured in Erlenmeyer flasks with the BBM medium. BBM stock solutions (per liter) contain 25 g sodium nitrate (NaNO_3_); 2.5 g calcium chloride dehydrate, (CaCl_2_.2H_2_O); 7.5 g magnesium sulfate heptahydrate (MgSO_4_.7H_2_O); 7.5 g potassium phosphate (K_2_HPO_4_); 17.5 g monopotassium phosphate (KH_2_PO_4_); 2.5 g sodium chloride (NaCl); 11.42 g boric acid (H_3_BO_3_); alkaline EDTA solution, acidified iron solution; trace metal solution containing 8.82 g zinc sulfate heptahydrate (ZnSO_4_.7H_2_O); 1.44 g manganese (II) chloride tetrahydrate (MnCl_2_.4H_2_O); 0.71 g molybdenum trioxide (MoO_3_); 1.57 g copper (II) sulfate pentahydrate (CuSO_4_.5H_2_O); and 0.49 g cobalt (II) nitrate hexahydrate (Co(NO_3_)2.6H_2_O)(Bold 1949). Cultures were incubated under fluorescent lamps illumination in a 12/12 dark/light cycle, at an irradiance level of 70 mol photons m^−2^ s^−1^ and room temperature. To monitor cell growth and quantify the different microalgae biomass calculation, cell density was measured by counting using a hemocytometer and a light microscope. Optical density was measured by the absorbance using a UV–Visible spectrophotometer (BioTek, Epoch, Gen5) at the wavelength of 600–680 nm. To calculate the dry weight of the samples (biomass per volume of culture (g/L), samples were centrifuged at 8000×*g*, 10 min and the biomass cake was dried at 60 °C overnight.

### Morphological and molecular characterization

During the culturing process capsule’s microalgae were identified by observation of morphological characteristics under a light microscope with negative staining. After elementary identification, the molecular analysis was further confirmed by sequencing of the 18S ribosomal RNA gene.

### sPS extraction

Dried microalgae's powder was subjected to acidic hydrolysis using hydrochloric acid (0.07%) at 90 °C for 4 h. After hydrolysis, the mixture was centrifuged (10,000×*g*, 10 min, 4 °C) and then the supernatant was precipitated with 95% ethanol (1:1) (v/v) with mechanical stirring and kept at 4 °C overnight. To obtain the polysaccharide-enriched fraction, the mixture was centrifuged (10,000×*g*, 10 min, 4 °C), collected, lyophilized, and stored at 4 °C for further study. The sPS extraction yield (%) was calculated as follows (Ale et al. 2012):

$${\text{sPS}}\,{\text{extraction yield }}\left( \% \right) = \,{{{\text{Dried crude sPS weight }}\left( {\text{g}} \right)} \mathord{\left/ {\vphantom {{{\text{Dried crude sPS weight }}\left( {\text{g}} \right)} {{\text{Powder weight }}\left( {\text{g}} \right) \times {1}00\% }}} \right. \kern-\nulldelimiterspace} {{\text{Powder weight }}\left( {\text{g}} \right) \times {1}00\% }}$$

### Determination of sPS total carbohydrates

Total carbohydrates of EPS were analyzed by the phenol–sulfuric acid method as described by Dubois et al. (1956). It is based on the production of furfural derivatives due to the dehydration of hydrolyzed saccharides with concentrated sulfuric acid. Thereafter, 5% phenol was added to the solution and incubated in the water bath for 10 min at 90 °C. After cooling to room temperature, the plate was read using a UV–Visible spectrophotometer (BioTek, Epoch, Gen5) at 490 nm. The calibration curve was created using known concentrations of D-glucose (0–0.2 mg/mL) and heparin sodium stock solution (0–5 mg/mL) as a reference standard.

### Determination of sPS sulfate content

The sulfate content measurement was carried out according to a BaCl_2_ gelatin method with some modifications (Dodgson and Price 1962). Briefly, the samples were hydrolysis in N-hydrochloric acid 1 M at 105–110 °C for 5 h. Then, 3% (w/v) trichloroacetic acid and barium chloride-gelatin reagent were added to the mixture, which was kept for 15–20 min at room temperature. The solution absorbance was measured at 360 nm (BioTek, Epoch, Gen5). The calibration curve was obtained using K_2_SO_4_ as a standard (55–550 µg/mL).

### HPLC and infrared spectroscopic analysis of sPS

The samples were hydrolyzed in 0.07% HCl for 4 h in 90 °C. The monosaccharides in the microalgae extract were quantified by HPLC on Eurokat H column (8 mm × 30 cm), using sulfuric acid 0.01 N (pH: 2.15) as the mobile phase (flow rate 0.6 mL/min) and refractive index detector, as described by Dokhani et al. (1988). The IR spectra of the microalgae extracts samples were recorded on Bruker Tensor 27 FTIR spectrometer between 400 and 4000 cm^−1^ (Baky et al. 2013).

### Antioxidant activity

#### DPPH radical scavenging assay

The 2, 2 diphenyl-1-picrylhydrazyl (DPPH) radicals were used to investigate the scavenging activity of sPS. The assay was carried out in a 96 well microplate with 200 µL of DPPH (2 Mm at ethanol) solution and sample. Different concentrations (0.5–2 mg/mL) were tested for each sample in triplicate. The plate was then covered and kept for 30 min in the dark at room temperature and then absorbance was read at 517 nm using a microplate reader (BioTek, Epoch, Gen5). The percentage of DPPH scavenging activity was calculated (Romano et al. 2009).

#### ABTS radical cation scavenging assay

The capability of sPS scavenging activities of 2,20-azino-bis (3-ethylbenzothiazoline-6-sulphonic acid) (ABTS) radical cation was determined with the method reported by Liu et al. with some modifications. After preparation of ABTS solution (absorbance: 0.7 ± 0.05 at 734 nm), the samples solutions (2, 1, 0.5, 0.1, and 0.05 mg/mL) were mixed with ABTS diluent. Then this mixture was kept in dark at room temperature for 6 min and the absorbance was measured at 734 nm in a microplate reader (BioTek, Epoch, Gen5). The scavenging rate (%) was used to evaluate the ABTS scavenging capacity of different sPS (Liu et al. 2016).

### Anticoagulant activity

Anticoagulant activities of EPS samples were evaluated using prothrombin time (PT) and activated partial thromboplastin time (APPT) assay. Control plasma samples were mixed with various EPS concentrations (0.05–2 mg/mL) and incubated at 37 °C for 60 s. The mixture and pre-warmed aPTT assay reagent were incubated at 37 °C for 2 min. Finally, pre-warmed calcium chloride (0.25 mol/L) was added and clotting time was recorded. In prothrombin time (PT) assay, control plasma was mixed with EPS samples in different concentrations (0.025–0.2 mg/mL). Then pre-warmed PT assay reagent was added and clotting time was recorded (Li et al. 2016). The heparin was used as standard (0–0.05 mg/mL).

### Statistical analysis

The carbohydrate and sulfate content data for microalgal strains were performed using a one-way analysis of variance (ANOVA, p < 0.05) with applying the Minitab 18 software. Another statistical analysis was carried out using excel 2019. All experiments were performed at least in triplicate and data were represented as mean ± standard deviation.

## Result

### Microalgae growth monitoring

**Fig. 1:**
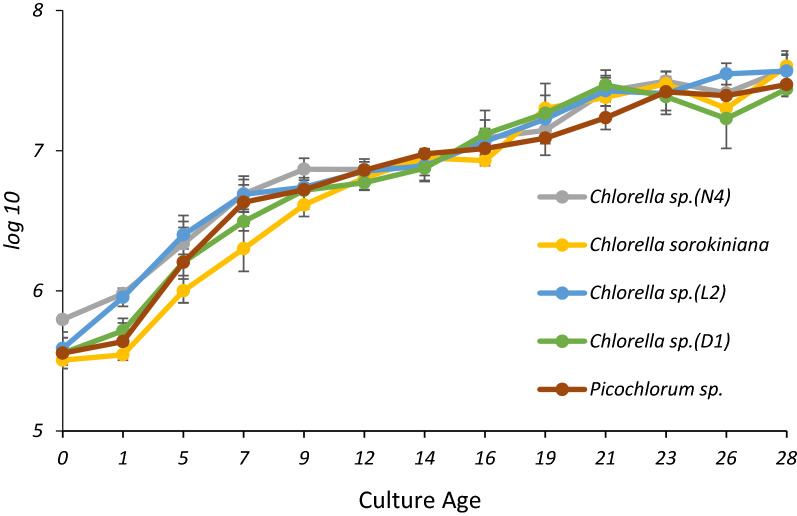
Comparison of cell density growth profile from different algal strains grown in BBM media

The microalgae growth was determined by optical density, cell counting and dry weight. The exponential and linear phases of growth occurred between 12 and 16th day. The highest growth percentage was observed at the exponential phase and then the growth decreases. The cultivation process was monitored up to 28 days and the stationary phase of isolated microalgae occurred between the 16th and 28nd day (Fig. 1). The cultivation period has been extended till 28 days because EPS is produced in the stationary phase.

### Microalgae morphological and molecular characterization

Morphological characters such as the presence of mucilaginous capsules surrounding microalgae cells were observed using light microscopy. The isolated microalgae were identified based on molecular studies using 18S rRNA analysis to phylogenetically identify EPS-producing microalgae strains. The partial sequences of the 18S rRNA gene of L_1_, N_4_, D_1_, and L_2_ (816 bp, 850 bp, 980 bp and 1216 bp) were submitted to GenBank, respectively. The result of the BLAST analysis of the rRNA sequence was shown in Table1.

**Table 1:** Phenotype of microalgae strains isolated from the Persian Gulf

	Empire	Kingdoms	Phyla	Classes	Orders	Families	Genera	Species	PTCC number	Accession number
L_1_	*Eukaryota*	*Plantae*	*Chlorophyta*	*Trebouxiophyceae*	*Chlorellales*	*Chlorellaceae*	*Chlorella*	*sorokiniana*	M-8011	OK570187
N_4_						*Chlorellaceae*	*Chlorella*	sp.	M-8013	OK663517
D_1_						*Chlorellaceae*	*Chlorella*	sp.	M-8010	OK663518
L_2_						*Chlorellaceae*	*Chlorella*	sp.	M-8012	OK572469
S_1_						*Chlorellales incertae sedis*	*Picochlorum*	sp.	M-6032^a^	OK572468

^a^Abolhasani et al. (2018)

### Carbohydrate and sulfate estimation

**Fig. 2:**
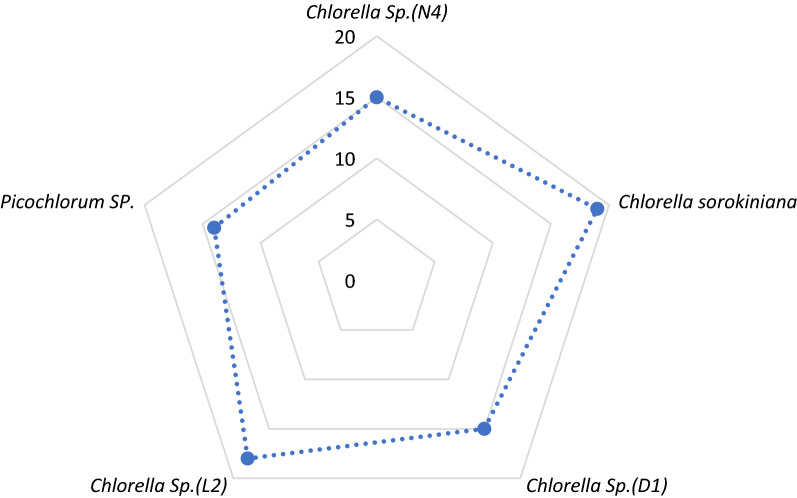
Microalgae extract yield percentage

As presented in Fig. 2, *C.* *sorokiniana* and *Chlorella *sp. *(L*_*2*_*)* showed the highest carbohydrate production. In this study yields of crude EPS produced by the microalgae strains ranged from 14 to 19% (w/w) using heated acid in 4 h at 90 °C.

**Fig. 3:**
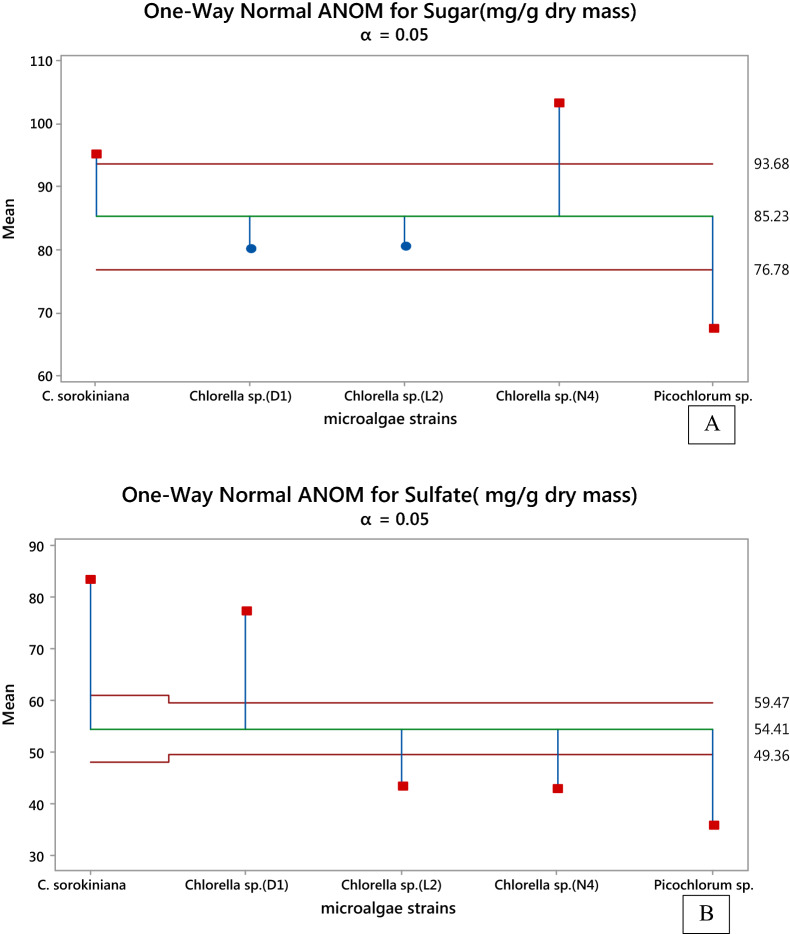
One-way normal Analysis of Mean (ANOM) for **A** sugar and **B** sulfate (mg/g dry biomass) in different strains (Alpha ≤ 0.05)

The carbohydrate contents in the EPSs were also found to vary greatly, ranging from 67 mg/g dry mass of *Picochlorum *sp. strain to 103 mg/g dry mass of *Chlorella *sp. *(N*_*4*_*)* strain. Data in Fig. 3(A) shown that *Chlorella *sp. *(N*_*4*_*)* and *C. sorokiniana* produced higher carbohydrate content compared to other strains. Although *Chlorella *sp.* (N*_*4*_*)* had a low crude EPS extract but possess higher content of the EPS sugar (103 mg/g dry extract) as compared to 95 mg/g dry mass of *C. sorokiniana*.

In all the studied species, the EPSs with different sulfate contents were obtained ranging from about 35 mg/g dry biomass produced by *Picochlorum *sp. to about 93 mg/g dry biomass produced by *C. sorokiniana* (Fig. 3).

**Fig. 4:**
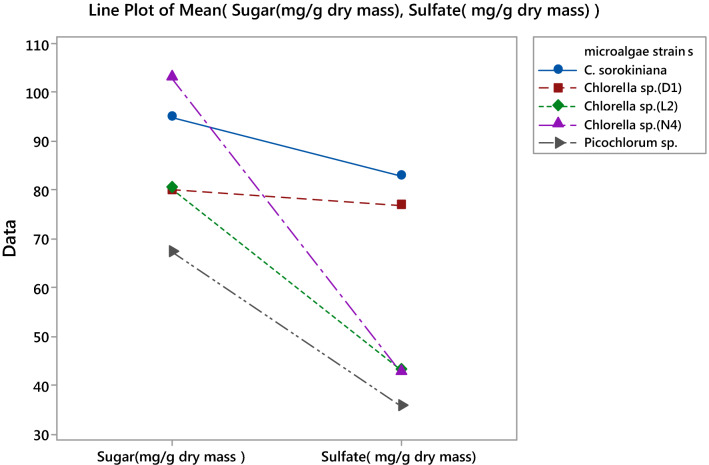
Comparison of strains in terms of sulfate and sugar content with an emphasis on high sulfate in lower sugar content in EPS extracted of various microalgae strains by line plot of the mean

As shown in Fig. 4, the highest sulfate content of sPS from *C. sorokiniana*, and *Chlorella* sp. *(D*_*1*_*)* were reported to 93 and 76 mg/g dry mass, respectively.

Also, in this study carbohydrate and sulfate content of the algal extract were evaluated and compared with heparin sodium, as standard. A comparative data for total carbohydrate and sulfate content was presented in Table2.

**Table 2:** Physio-chemical characteristics of biomass microalgae strains and EPS extracted by comparing heparin sodium and glucose standard (Value expressed as mean ± SD)

	Genus	Weigh g/L (dry mass)	Yield (%) of extract from dry microalgae	Carbohydrate Content (mg/g) from crude extract (Glucose as standard)	Sulfate ester content (mg/g) From crude extract (K_2_SO_4_ as standard)	Rate of sulfate/sugar	Carbohydrate Content (Equivalent heparin sugar(mg)/g dry biomass	Sulfate ester content (Equivalent heparin sulfate(mg)/g dry biomass
N_4_	*Chlorella* sp.	0.84	15 ± 0.02	103.12 ± 2.52	42.65 ± 0.86	0.41	768 ± 18	85 ± 5
L_1_	*Chlorella sorokiniana*	0.51	19 ± 0.05	95.06 ± 6.78	93.73 ± 5.95	0.98	709 ± 50	338 ± 36
L_2_	*Chlorella* sp*.*	0.86	15 ± 0.02	80.54 ± 1.66	43.21 ± 3.68	0.53	600 ± 12	94 ± 16
D_1_	*Chlorella* sp.	0.59	18 ± 0.05	80.04 ± 5.84	76.95 ± 2.35	0.96	597 ± 43	245 ± 14
S_1_	*Picochlorum* sp.	0.55	14 ± 0.01	67.40 ± 7.31	35.79 ± 2.29	0.53	502 ± 54	80 ± 6

In this respect, *Chlorella *sp. *(N*_*4*_*)* and *C. sorokiniana* with 768 ± 18 and 709 ± 50 carbohydrate content equivalent heparin sugar mg/g dry biomass, presented the highest sugar content. But *C. sorokiniana* and *Chlorella *sp. *(D*_*1*_*)* contained more equivalent heparin sulfate. For example, the carbohydrate content in 1 g of *C. sorokiniana* dry extract was equivalent to 0.7 g heparin sugars content. According to the results, the sugar content of heparin equivalent in sulfated polysaccharides extracted from the studied strains was estimated to be 50–77% (Table 2). The sugar content of sPS was measured also using D-glucose as standard.

### Fourier transform infrared (FT-IR) characterization and monosaccharide composition of the sPS

**Fig. 5:**
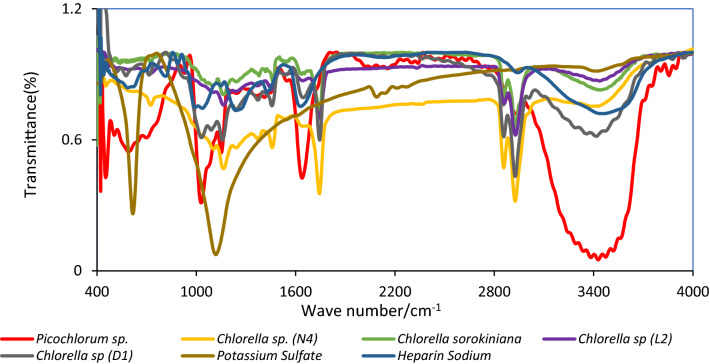
FT-IR spectra of sPS extracted by comparing heparin and potassium sulfate as standard between 400 and 4000 cm^−1^

The FTIR spectra characters of polysaccharides and heparin sodium as standard was presented in Fig. 5. A total of 11 monosaccharides (mannose, glucosamine, trehalose, glucose, galactose, xylose, fructose and ribose) and oligosaccharides (sucrose, maltose and raffinose) were used as standard. The HPLC chromatogram of the crude sPS exhibited in Table 3.

**Table 3:** Monosaccharide composition of the polysaccharides extracted from microalgae strains

sPS extracted from microalgae	t_R_(min) 8.6	t_R_(min) 6.8	t_R_(min) 7.7	t_R_(min) 10.6	t_R_(min) 9.9	t_R_(min) 10.8	t_R_(min) 10.6	t_R_(min) 10.6	t_R_(min) 12	t_R_(min) 8.4	t_R_(min) 8.4
	Sucrose (mg/mL)	Glucosamin (mg/mL)	Raffinose (mg/mL)	Mannose (mg/mL)	Glucose (mg/mL)	Fructose (mg/mL)	Xylose (mg/mL)	Galactose (mg/mL)	Ribose (mg/mL)	Trehalose (mg/mL)	Maltose (mg/mL)
*Chlorella* sp. *(N*_*4*_*)*	1.95	4.5	–	–	0.38	–	–	–	–	–	–
*Chlorella sorokiniana*	123.66	2.5	0.08	–	0.25	–	–	–	–	–	–
*Chlorella* sp. *(L*_*2*_*)*	2.27	5.25	–	–	0.76	–	–	–	–	–	–
*Chlorella* sp. *(D*_*1*_*)*	1.2	2.7	–	–	–	–	–	–	–	–	–
*Picochlorum* sp.	1.06	2.38	–	–	0.5	–	–	–	–	–	–

### Radical scavenging activities

**Fig. 6:**
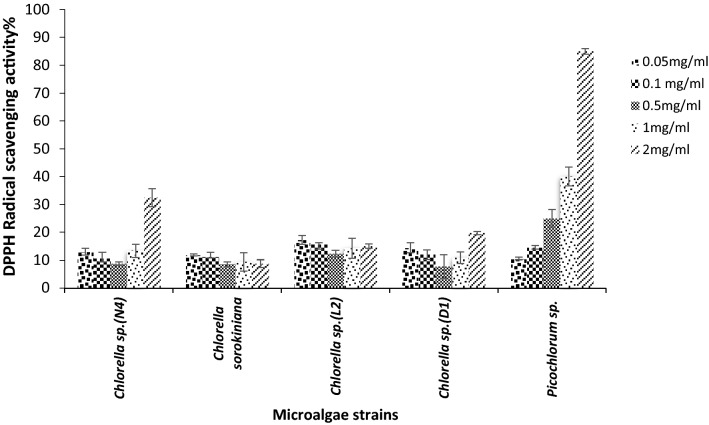
Antioxidant activity, 1,1-diphenyl-2-picrylhydrazyl (DPPH) radicals scavenging activity by sPS extracted of various microalgae strains

The DPPH and ABTS colorimetric methods were used to assess the possible antioxidant potential of sPS. The scavenging DPPH radical’s activity of sPS was determined. As shown in Fig. 6 some sulfated polysaccharides samples revealed apparent inhibition activities on DPPH radicals. Our results exhibited that the DPPH scavenging activity of *Picochlorum sp.* sulfated polysaccharides was significantly higher than other extracted polysaccharides (p < 0.05).

**Fig. 7:**
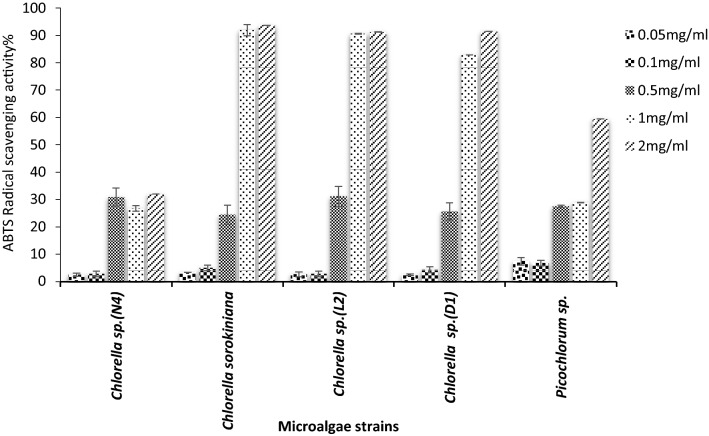
Antioxidant activity, ABTS radicals scavenging activity by sPS extracted of various microalgae strains

The ABTS radical scavenging activities of sPS extracted from five microalgae strains at different concentrations (0.05–2 mg/mL) were evaluated (Fig. 7). The ABTS radical scavenging of sPS of all strains except *Chlorella *sp. *(N*_*4*_*)* may be due to the least sulfate/sugar ratio, reached to higher than 50% activity at 1 mg/mL.

### Anticoagulant activities

The anticoagulant activity of sPS and heparin as control were measured using PT and aPTT assay. As shown in Table 4, *C. sorokiniana* sPS prolonged clotting time more than 38 s at 10 µg/mL in aPTT test. The PT activity by *C. sorokiniana* and *Chlorella* sp. *(N*_*4*_*)* sPS at 200 µg/mL increased clotting time (up to 14 s).

**Table 4:** Analysis of anticoagulant activity by aPTT and PT assay on Sps

	µg/mL	aPTT(s)	T_1_/T_0_^a^	µg/mL	PT(s)	T_1_/T_0_^a^
*Chlorella* sp. *(N*_*4*_*)*	50	24.19 ± 0.81	0.71 ± 0.03	25	12.88 ± 0.02	0.98 ± 0.008
	75	23.83 ± 0.31	0.70 ± 0.009	50	12.89 ± 0.14	0.98 ± 0.005
	100	38.56 ± 1.99	1.18 ± 0	75	12.90 ± 0.09	0.99 ± 0.003
	200	19.41 ± 2.19	0.57 ± 00.01	100	13.04 ± 0.007	1 ± 0.0002
	–		–	200	14.52 ± 0.5	1.11 ± 0.01
*Chlorella sorokiniana*	50	26.79 ± 1.28	0.79 ± 0.03	25	11.90 ± 0.28	0.91 ± 0.01
	75	23.08 ± 0.55	0.68 ± 0.01	50	12.96 ± 0.07	0.99 ± 0.002
	100	35.83 ± 1.06	1.05 ± 0.03	75	13.3 ± 0.82	1.05 ± 0.004
	200	32.56 ± 2.19	0.98 ± 0.03	100	13.01 ± 0.04	0.99 ± 0.001
	–		–	200	14.26 ± 0.34	1.09 ± 0.01
*Chlorella* sp. *(L*_*2*_*)*	50	23.41 ± 0.94	0.69 ± 0.02	25	11.88 ± 0.16	0.91 ± 0.006
	75	16.53 ± 1.45	0.48 ± 0.04	50	12.91 ± 0.09	0.99 ± 0.003
	100	36.78 ± 1.24	1.08 ± 0.03	75	12.54 ± 0.23	0.96 ± 0.008
	200	18.65 ± 0.55	0.55 ± 0.01	100	13.56 ± 0.27	1.04 ± 0.01
	–		–	200	13 ± 0.07	0.99 ± 0.002
*Chlorella* sp. *(D*_*1*_*)*	50	19.69 ± 0.01	0.58 ± 0.006	25	12.45 ± 0.44	0.95 ± 0.01
	75	27.89 ± 0.43	0.82 ± 0.01	50	13.75 ± 0.54	1.07 ± 0.03
	100	32.05 ± 1.20	0.94 ± 0.03	75	12.70 ± 0.36	0.97 ± 0.01
	200	33.74 ± 0.68	0.99 ± 0.02	100	13.43 ± 0.08	0.99 ± 0.03
	–		–	200	13.82 ± 0.53	1.06 ± 0.02
*Picochlorum* sp.	50	31.69 ± 1.87	0.93 ± 0.05	25	12.36 ± 0.11	0.94 ± 0.004
	75	21.3 ± 1.20	0.62 ± 0.03	50	11.26 ± 0.53	0.86 ± 0.02
	100	25.07 ± 0.47	0.74 ± 0.01	75	12.85 ± 0.21	0.98 ± 0.008
	200	21.49 ± 0.51	0.63 ± 0.01	100	12.62 ± 0.49	0.96 ± 0.01
	–		–	200	13.54 ± 0.06	1.03 ± 0.022

HEP 5 µg/mL; aPTT: 60.05 ± 0.007 s; Control: 33.87 ± 0.45 s and HEP 25 µg/mL; PT: 13.69 ± 0.2 s; Control: 13.03 ± 0.2 s. Data expressed as mean ± standard deviation (n = 3)

^a^Clotting time in presence sPS(T_1_) and absence (T_0_)

sPS extracted from *Chlorella *sp. *(N*_*4*_*)* with the lowest sulfate/sugar ratio showed the highest PT activity, but despite high sulfate/sugar ratio in *Chlorella *sp. *(D*_*1*_*)* sPS, no anticoagulant activity was observed.

## Discussion

Five microalgae strains isolated from the Persian Gulf were evaluated for biomass, polysaccharides production and their biological activities. The production of microalgal EPSs generally is related to the microalgae cultivation condition and is different from species to species (Delattre et al. 2016). Also, the extraction method has a significant effect on the extraction yields of microalgae polysaccharides (Yuan et al. 2020). According to our data the heated acid hydrolysis extraction method of *Chlorella *sp. polysaccharide yields ranged from14 to 19% which is in agreement with that (18%) of 80–90 °C water extraction method from the *Chlorella* source (Lai and Sun 2017). Also, Sui et al. (2012) study showed an extracted polysaccharide yield ranged from 13 to 19% from *Chlorella* sp. with hot water incubation (90 °C, 30 min). While the other study reported a crude polysaccharide yield of approximately 9.62 ± 0.11% dry weight of *Chlorella-Arc* in the 88 °C and 3 h incubation (Song et al. 2018). The significant effect of the temperature on polysaccharide yield is related to the breaking of the internal and external hydrogen bonds of biopolymer and increasing the solubility of polysaccharides and their diffusion in water (Kumar and Fotedar 2009).

Likewise, the acid heated extraction EPS yield (19%) for C. *sorokiniana* in this study remained higher than those obtained by Suárez et al. for the *C.* *sorokiniana* (15%) by using hot water extraction (Suárez et al. 2005). The highest rate of sulfate/sugar, 0.98 and 0.96 were observed for *C. sorokiniana* and *Chlorella *sp. *(D*_*1*_*),* respectively (Table 2). But this ratio is slightly lower than El-Naggar et al. presented results (sulfate/sugar ratio:1.2) for *Chlorella vulgaris* polysaccharides extract (El-Naggar et al. 2020). Our findings were consistent with those obtained by Nishino and Nagumo who reported the sulfate/sugar ratios of macroalgae *Ascophyllum nodosum* and *Fucus vesiculosus* as 0.93 and 0.90, respectively (Nishino and Nagumo 1992).

HPLC analysis of extracted EPS under 0.07% acid hydrolysis at 90 °C indicated that it is composed of different sugars in the form of monomer (glucose), dimer (sucrose), or trimer (raffinose). Sucrose was dominant in the sulfated polysaccharide extracted from *C. sorokiniana* acid hydrolysate. Glucosamine was the most abundant monosaccharide in the *Chlorella *sp. *(L*_*2*_*)* and *Chlorella *sp. *(N*_*4*_*),* respectively*.* Detection of amino sugar in *Chlorella vulgaris* cell wall was also reported previously by Ortiz-Tena et al. (2016). In addition, they revealed that detected glucosamine in microalgae is most probably part of chitin-like polymer available as N-acetyl glucosamine in the cell wall (Ortiz-tena et al. 2016). In our study small amount of raffinose were observed in *C. sorokiniana* extracellular sugar. According to our knowledge, only one study reported accumulation of raffinose in unicellular *Chlorella vulgaris* after cold shock (Salerno and Pontis 1989). The culture condition and hydrolysis procedure including acid concentration, hydrolysis temperature and incubation time affect the microalgae monosaccharide’s profile. The effect of culture conditions and polysaccharides hydrolysis procedure including acid concentration, temperature and incubation time, on the microalgae monosaccharides profile was confirmed previously (Schulze et al. 2017).

The absorbance at wavenumbers 3440 cm^−1^ OH and 1200–800 cm^−1^ shows the IR spectrum of carbohydrates (McCann et al. 1992). The absorption band at around 3400–4450 cm^−1^, may be attributed to O–H in *C. sorokiniana, Chlorella *sp. *(D*_*1*_*), Chlorella *sp. *(L*_*2*_*)* and *Picochlorum *sp. stretching and standard spectra of heparin sodium. The band at 2924 cm^−1^ was observed in *C. sorokiniana, Chlorella *sp. *(D*_*1*_*)* and heparin sodium was the characteristic peak of C–H of the CH_2_ group. The band found at1440–1460 cm^−1^ was due to symmetric stretch vibration of COO– and C–O within COOH in sPS from *C. sorokiniana*, *Chlorella *sp.* (N*_*4*_*)* and *Chlorella* sp. *(L*_*2*_*).* The peak at 1742 cm^−1^ is derived from ester carbonyl groups stretching band in *Chlorella *sp. *(D*_*1*_*)*, *C. sorokiniana*, *Chlorella *sp. *(L*_*2*_*)* and *Chlorella *sp. *(N*_*4*_*)*. 1641 cm^−1^, corresponding to the carbonyl group of a carboxylic acid group of uronic acid in *Chlorella *sp. *(L*_*2*_) and heparin sodium. The band at 1157 cm^−1^ is related to the C–O stretch in phenolic compounds available in *Chlorella *sp. *(D*_*1*_*).* The peaks identified 1025 cm^−1^ in *C. sorokiniana* correspond to the –CH_2_OH groups and to the C–OH stretching and bending of the C–OH moiety of carbohydrates indicate a rich glycogen superficial layer. Carbohydrate FTIR analysis in this study was in agreement with that found by Dore et al. (2013), Guo et al. (2018), De Castro et al. (2018), Fernando et al. (2017)*,* Lai and Sun (2017)*,* Alvares et al. (2021) and Balan et al. (2019). The intense peak was found in all sPS extracted from all microalgae except *Picochlorum *sp. at 1095–1164 cm^−1^, indicating stretching vibration of the 1070–1172 cm^−1^ in K_2_SO_4_ (Periasamy et al. 2009) and suggesting the presence of sulfate group.

The correlation of the polysaccharide structures with their biological function fulfilled the bioactivity of the exopolysaccharides produced by these strains. The maximum DPPH scavenging activities (85%) were observed at the concentration of 2.0 mg/mL for *Picochlorum *sp. sPS. The hydrogen atom in the hydroxyl group available in *Picochlorum *sp. sPS (FTIR data) leads to the stronger hydrogen atom-donating capacity in DPPH scavenging assays. Many researchers have investigated the in vitro and in vivo antioxidant activities of microalgae polysaccharides. The 16-polysaccharide extracted from *C. pyrenoidosa* by Hu et al. (2007) displayed significant anti-radical activities, varying from 29.67 ± 0.29% to 54.16 ± 4.49% at 10 mg/mL (Hu et al. 2007). In our study notable antioxidant activity was not observed in sPS extracted from microalgae *C. sorokiniana* (*C. pyrenoidosa*) at tested concentrations (0.05–2 mg/mL). Our finding was in keeping with many reports on the antioxidant activities of sPS extracted from algae. The evaluation of antioxidant activities of exopolysaccharides from *Chlorella zofingiensis (EPS-CZ)*, *C. vulgaris (EPS-CV)* and *C. pyrenoidosa (EPS-CP)* demonstrated DPPH radical scavenging activities. At the concentration of 3.0 mg/mL, the EPS-CV and EPS-CZ inhibited 59.6% and 71.5% of DPPH radicals, respectively. The EPS-CP had 55.2% of DPPH radical scavenging activity at the concentration of 2.0 mg/mL (Zhang et al. 2019). According to Zhang et al. (2013) report water and alkali extracted polysaccharides from *Enteromorpha linza* showed over 83 and 89% DPPH scavenging activity, respectively at 3.61 mg/mL concentration. The high DPPH scavenging activity, 73%, at 160 µg/mL of extracted sPS from *Gracilaria corticate* and the IC_50_ value of 32 µg/mL for *Nostoc carneum* exopolysaccharide has been reported (Seedevi et al. 2016; Mervat et al. 2015). Also, the DPPH radical scavenging effect of crude polysaccharide from *C. pyrenoidosa* in concentration 10mg/mL was reported as 54.16 ± 4.49% (Hu et al. 2007). The result of the present study indicated that due to the high electrons donating ability of sulfated polysaccharides of *C. sorokiniana*, *Chlorella *sp. *(L*_*2*_*)* and *Chlorella *sp. (*D*_*1*_), the ABTS radical scavenging activity was increased to > 82% at the concentration of 1 mg/mL.

It is interesting to determine the chemical constituents and scavenging activity relationship for sPS extracted from microalgae. The sulfated polysaccharides from *Chlorella *sp. *(L*_*2*_*)* and *Picochlorum *sp. with similar sulfate/sugar ratio showed potent radical scavenging activities 91% in ABTS and 85% in DPPH scavenging activity, respectively.

The hypothesis of the relationship between the free radical scavenging capability of EPS and the existence of functional groups such as C=O, –COOH, –O– and –OH in its structure due to donating electron activity is well accepted (Sun et al. 2015).

Based on the FTIR spectrum result, it is possible that high ABTS radical scavenging in *C. sorokiniana*, *Chlorella *sp. *(L*_*2*_*)* and *Chlorella *sp. (D_1_) sPS samples were due to the existence of the carbonyl group of a carboxylic acid group of uronic acid and the hydrogen atom in the hydroxyl group. Evaluating the results acquired for sPS extracted from four *Chlorella* species, sPS *C. sorokiniana*, *Chlorella *sp. *(D*_*1*_*)* and *Chlorella *sp. *(L2)* showed higher antioxidant activity than *Chlorella *sp.* (N*_*4*_*).* The sPS extracted from *chlorella* strains except for *Chlorella *sp.* (N*_*4*_*)* had relatively strong ABTS radical trapping, and the scavenging activity was 90% when the polysaccharide concentration was 2 mg mL^−1^. Since the electron transfer reaction in polysaccharides provides the hydrogen or electrons required to form stable molecules from free radicals (Wang et al. 2022). This result suggested that ABTS scavenging capacity may be due to the presence of the broad peak vibrations of the OH group (3200–3500 cm^−1^) in *C. sorokiniana*, *Chlorella *sp. *(D*_*1*_*) and Chlorella *sp. *(L2).* Low potency of the *Chlorella *sp. *(N*_*4*_*)* strain to inhibit free radicals was associated with the lack of this spectrum (FTIR spectrum data). Therefore since the ABTS assay evaluated non-physiological free radicals (Wang et al. 2022), it may be indicated sPS extracted from *C. sorokiniana*, *Chlorella *sp. *(D*_*1*_*)* and *Chlorella *sp. *(L2)* may directly scavenge free radicals.

ABTS scavenging activity of sPS extracted in the current study was higher than the scavenging activity of the previously reported study. According to Chen et al. (2018) study, the concentration of 4 mg/mL of AnPS polysaccharide fraction extracted from *Ascophyllum nodosum* had the highest ABTS radical scavenging activity (70%) among three different fractions.

Antioxidant activity is related to the molecular weight, sulfate content and position of a sulfate group, different type of the major sugar unit, glycosidic branching and glycoprotein residuals of polysaccharides (Wang et al. 2013; Wang et al. 2018; Chu, 2011; Qi et al. 2005). There is a correlation between uronic acids, the reducing and nonreducing end contents and antioxidant activities in polysaccharides (Wang et al. 2013; Wang et al. 2018).

It was observed that aPTT and PT activity of *C. sorokiniana* and *Chlorella *sp. *(N*_*4*_*)* sPS were lower than heparin. Heparin quickly increased the aPTT and PT times; the clotting time was more than 60 s at 5 µg/mL for aPTT and 13.69 s at 25 µg/mL for PT. Therefore, position to obtain the same effect as with heparin high concentration of sPS were required. By our results, five times lower anticoagulant activity in aPTT assay was reported for sPS purified from *Arthrospira platensis* than heparin (Majdoub et al. 2009). Also, the anticoagulant activity of two sPS extracted from *Monostroma nitidum* was reported to be weaker than heparin (Mao et al. 2008).

According to some research, in addition to sulfate concentration, the differences in the structural variation of sPS such as the distribution pattern and position of sulfate and chemical composition mainly the monosaccharide units could affect the sPS anticoagulant activity (Li et al. 2015; Yang et al. 2002; De Jesus Raposo et al. 2013). As can be seen from previous studies, the abundance of glucuronic acid branches in *Cladosiphon okamuranus* fucoidan, adversely affected the aPPT assay (Cumashi et al. 2007).

Conceptually, polysaccharides with in vitro low anticoagulant effects could be assumed as antithrombotic agents (Liet al. 2015; Mourão, 2015). In this regard, the tested sPS with poor anticoagulant activity may exhibit antithrombin activity in bleeding disorder which requires further study.

In total, the difference between the sulfate heteropolysaccharides extracted from the three *chlorella* strains in this study lies in their performance. For the anticoagulant effect, *C. sorokiniana* and *Chlorella *sp.* (N*_*4*_*)* showed higher activity than other chlorella strains. One explanation for this may be due to similar carbohydrate content equivalent heparin sugar (mg)/g dry biomass in sPS extracted of *C. sorokiniana* and *Chlorella *sp. *(N*_*4*_*).* Moreover, the content of sulfate esters plays an important role in sPS bioactivities. Interestingly, *Chlorella *sp. *(N*_*4*_*)* with a lower sulfate/sugar ratio (0.41) showed lower antioxidant activity*.* Whereas *Chlorella *sp. *(D1)* with sulfate/sugar ratio 0.53 following *C. sorokiniana* and *Chlorella *sp. *(L2)* with sulfate/sugar ratios 0.98 and 0.94, respectively*,* presented a high antioxidant effect. As a result, the finding of this study, suggest that antioxidant and anticoagulant activity of sPS was related to the sulfate ester content. This result indicates that some of the sPS could be a potential resource of natural antioxidants for further investigation in the food industry. So commercial development of this natural product can lead to the extension of functional food additives. Meanwhile, the *C. sorokiniana* sPS with anticoagulant and antioxidant activities could be a promising anticoagulant candidate with lower bleeding risk in stroke and heart ischemic disease.

## Data Availability

The data that support the findings of this study are available from the corresponding author upon reasonable request.

## Abbreviations

EPS: Exopolysaccharides
sPS: Sulfated polysaccharide
ABTS: 2,2′-Azino-bis(3-ethylbenzthiazoline-6-sulfonate)
DPPH: 1,1-Diphenyl-2 picrylhydrazyl
PT: Prothrombin time
APPT: Activated partial thromboplastin time
